# Exploring the Potential Mechanism of Qi-Shen-Di-Huang Drug Formulary for Myasthenia Gravis (MG) based on UHPLC-QE-MS Network Pharmacology and Molecular Docking Techniques

**DOI:** 10.1155/2022/7416448

**Published:** 2022-10-03

**Authors:** Yibin Zhang, Tianying Chang, Qi Lu, Yingzi Cui, Dongmei Zhang, Baitong Wang, Peng Xu, Jing Lu, Jinhui Ma, Zhiguo Lv, Jian Wang

**Affiliations:** ^1^Department of Neurology, The Affiliated Hospital to Changchun University of Traditional Chinese Medicine, Changchun, China; ^2^Evidence Based Office, The Affiliated Hospital to Changchun University of Traditional Chinese Medicine, Changchun, China; ^3^Medical Insurance Office, The Third Affiliated Clinical Hospital of Changchun University of Traditional Chinese Medicine, Changchun, China; ^4^Scientific Research Office, The Affiliated Hospital to Changchun University of Traditional Chinese Medicine, Changchun, China; ^5^Research Center of Traditional Chinese Medicine, The Affiliated Hospital to Changchun University of Traditional Chinese Medicine, Changchun, China; ^6^Department of Health Research, Evidence, and Impact, McMaster University, Hamilton ON, Canada

## Abstract

Myasthenia gravis (MG) is a rare and refractory autoimmune disease, and Qi Shen Di Huang (QSDH) drug formulary is an in-hospital herbal decoction with proven clinical efficacy in treating MG. Currently, most of the research on the QSDH drug formulary has concentrated on its clinical efficacy, and there is a lack of systematic study on the material basis. The active compounds and their mechanism of action have not been entirely determined. Therefore, this study sought to identify the active compounds in the QSDH drug formulary and analyze the key targets and potential mechanisms. We used ultra-performance liquid chromatography Q Exactive-mass spectrometry (UHPLC-QE-MS) and Traditional Chinese Medicine Systems Pharmacology Database and Analysis Platform (TCMSP) database to identify and screen 85 active ingredients corresponding to 59 potential targets (17 herbs) associated with myasthenia gravis, and further identified AKT1 as the primary core target and the PI3K/AKT signaling pathway as the most substantial enriched pathway. Molecular docking and UPLC-MS analysis identified quercetin, luteolin, wogonin, kaempferol, laccasein, and epigallocatechin gallate are the core compounds of the QSDH drug formulary. In vivo rat studies showed that the QSDH drug formulary reduced Lennon's clinical score and decreased acetylcholine receptor antibody levels in peripheral blood rats with experimental autoimmune myasthenia gravis. In addition, the QSDH drug formulary downregulated P-PI3K/PI3K and P-Akt/Akt protein expression. Collectively, these findings describe the role and potential mechanism of the QSDH drug formulary in the treatment of MG, which exerts potential value by acting on AKT targets and regulating the PI3K/AKT signaling pathway and providing a theoretical reference for QSDH drug formulary application in the clinical treatment of MG.

## 1. Introduction

Myasthenia gravis (MG) is an autoimmune disease with lesions in the postsynaptic membrane of the neuromuscular junction (NMJ). Pathogenic antibodies include the common acetylcholine receptor antibody (AChR-ab) and the less common muscle-specific receptor tyrosine kinase antibody (Musk-ab), and the low-density lipoprotein receptor-related protein4 antibody (LRP4-ab) blocks the aggregation of acetylcholine receptors (AChR) and disrupts the function of AChR and the signaling between NMJ [1]. Fatigue and fluctuating muscle contraction are the core clinical symptoms, which may involve skeletal muscles throughout the body, including the eyes, medulla oblongata, respiratory, and extremity muscles. The clinical presentation also varies depending on the autoantibody type and a thymoma's presence [2]. The global prevalence of MG is (150∼250)/million, and the prevalence of MG in China is about 0.68/per 100,000, slightly higher in women than in men. The whole body's skeletal muscle can be involved, and the symptoms are “light in the morning and heavy in the evening,” aggravated by activity and relieved by rest [3]. The treatment of MG is still mainly composed of cholinesterase inhibitors, glucocorticoids, immunosuppressants, intravenous immunoglobulins, and plasma exchange but traditional Chinese medicine has unique advantages in the treatment of MG, improving clinical efficacy while reducing the dosage and side effects of western drugs and improving the immune balance of the body [4].

The QSDH drug formulary is based on the theory of “Fu Xie” and “Nao Sui” which proposes that the fundamental mechanism of this disease is “a deficiency of spleen and kidney and deficiency of brain marrow,” and the treatment should be essential in the basic principle of “JianPi YiQi BuSui” [5]. The metabolic profile of TCM is the key to pharmacological research and clinical application. QSDH drug formulary consists of 17 herbs, including Astragalus, Radix Codonopsis, Atractylodes, Radix et Rhizoma, and others.

**Figure 1:**
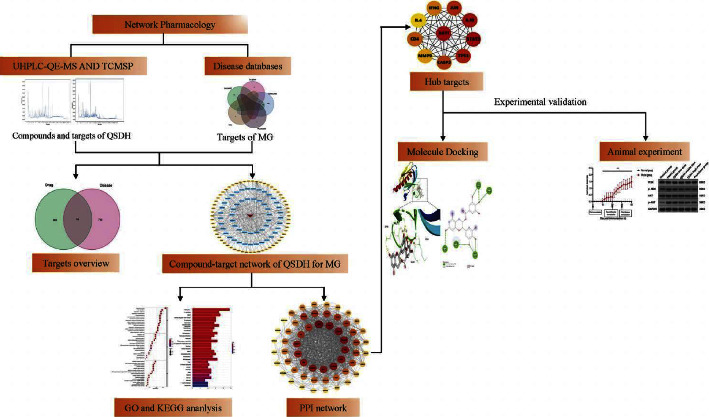
Outline of the study. Exploring the potential mechanism of Qi-Shen-Di-Huang drug formulary for myasthenia gravis (MG) based on UHPLC-QE-MS network pharmacology and molecular docking techniques.

The current research on the QSDH drug formulary is mainly focused on clinical efficacy, lacking systematic material basis research, in-depth research on its overall chemical composition, the material basis of pharmacological efficacy, activity, and its active ingredients and specific mechanisms not fully understood. TCM research needs some new approaches and various network technologies have been evolving with the development of the information age. Cyberpharmacology has recently been an emerging discipline based on systems biology, multidirectional pharmacology, and proteomics. It combines pharmacology and information networks to construct molecular biological networks of diseases and drugs by analyzing the whole network to elucidate the pharmacological effects of drugs and pathogenesis of diseases through high-throughput histological data analysis and search of network databases [6]. We can directly identify drug and disease targets through network pharmacology from a large amount of data and understand the mechanisms and pathways [7]. Ultrahigh-pressure liquid chromatography coupled with high-resolution mass spectrometry has the advantages of solid separation and structure identification, which can reasonably predict the structure of unknown compounds. The combination of UHPLC-MS and network pharmacology has dramatically promoted the novel pharmacological research and drug development of TCM. Therefore, in our study (Figure 1), we used UHPLC-QE-MS to identify the active ingredients of the QSDH drug formulary and then screened the potential targets of action of the QSDH drug formulary for treating MG by network pharmacology. Finally, molecular docking techniques and animal experiments to validate the key targets and pathways of the QSDH drug formulary and its essential substances acting on MG.

## 2. Materials and Methods

### 2.1. Experimental Reagents and Apparatus

Methanol (CAS 67-56-1, CNW Technologies). Acetonitrile (CAS 75-05-8, CNW Technologies). L-2-chlorophenyl alanine (CAS 103616-89-3, Shanghai Hengbai Biotechnology Co., Ltd.). QSDH drug formulary (Jilin Yatai Yongan Tang Pharmaceutical Co., Ltd., lot 210303T). Prednisone acetate tablets (Shanghai ShangPharma Xinyi Pharmaceutical Co., Ltd., Lot No. H31020675). Rat-derived AchR*α* subunit 97–116 peptide fragment (Suzhou QiangYao Biotechnology Co., Ltd., China). Complete Fuchsin adjuvant (CFA), incomplete Fuchsin adjuvant (IFA), (Sigma-Aldrich, lot F5881, F5506, respectively). Dried powder of *Mycobacterium tuberculosis* H37RA (Difco Bacto, USA, lot 231141). Rat antiacetylcholinesterase receptor antibody (AchR-Ab). Enzyme-linked immunosorbent assay (ELISA) kit (Enzyme Immunity, Inc., Lot No. MM-70967R1). Phosphate buffer solution (PBS), (Thermo Fisher Scientific, Item 003002). Phosphatidylinositol 3-kinase (PI3K) antibody, (Abcam, Inc., Item No. Ab 223792). Phosphatidylinositol 3-kinase (p-PI3K) antibody, (Abcam, ab 182651). Phosphorylated protein kinase B (p-Akt) antibody, (Abcam, item #ab38449). Protein kinase B (Akt) antibody (Cell Signaling Technology, Inc., item #4691). Glyceraldehyde-3-phosphate dehydrogenase (GAPDH) internal reference antibody (Proteintech, item #10494-1-AP). RIPA lysate (strong), protein prestain marker, BCA protein quantification kit (P0013 B, P0068, P0010S, respectively). Ultrahigh-performance liquid chromatograph (Model 1290 UPHLC, Agilent). High-resolution mass spectrometer (model Q Exactive focus). Infinite M200 pro multifunctional enzyme standard (Tecan, Switzerland, model InfiniteM200Pro). Power Pac type electrophoresis instrument (Bio-Rad, USA). Fluor chem E alpha chemiluminescence gel imaging system (Protein Simple, USA). Protein Simple.

### 2.2. Metabolites Extraction

The samples were crushed with a mixer mill for 30 s at 65 Hz. 100 mg of sample was added to 500 *μ*L of an extracted solution containing 1 *μ*g/mL of internal standard and dissolved in 80% methanol. After the 30s vortex, the samples were homogenized at 45 Hz for 4 min and sonicated for 1 hour in an ice-water bath. After 1 hour in −40°C, the sample was centrifuged at 12000 rpm for 15 min at 4°C. Finally, the supernatant was obtained and put in a fresh 2 mL tube for LC-MS/MS analysis, taking 40 *u*l from each sample and pooling it as QSDH samples.

### 2.3. LC-MS/MS Conditions

LC-MS/MS analysis was performed on an Agilent ultra-high performance liquid chromatography 1290 UPLC system with a Waters UPLC BEH C18 column (1.7 *μ*m 2.1*∗*100 mm). The flow rate was set at 0.4 mL/min, and the sample injection volume was set at 5 *μ*L. The mobile phase consisted of 0.1% formic acid in water (A) and 0.1% in acetonitrile (B). The multistep linear elution gradient program was as follows: 0–3.5 min, 95–85% A. 3.5-min, 85–70%A. 6–6.5, 70–70% A. 6.5–12 min, 70–30% A. 12–12.5 min, 30–30% A. 12.5–18 min, 30–0% A. 18–25 min, 0–0% A. 25–26 min, 0–95% A. 26–30 min, 95–95% A.

A Q Exactive focus mass spectrometer coupled with an Xcalibur software was employed to obtain the MS and MS/MS data based on the IDA acquisition mode. During each acquisition cycle, the mass range was from 100 to 1500, the top three of every cycle were screened, and the corresponding MS/MS data were further acquired. Sheath gas flow rate: 45 Arb, Aux gas flow rate: 15 Arb, capillary temperature: 400°C, full ms resolution: 70000, MS/MS resolution: 17500, collision energy: 15/30/45 in NCE mode, spray voltage: 4.0 kV (positive) or −3.6 kV (negative).

### 2.4. QSDH Drug Formulary Screening of Active Ingredients and Targets of Action

According to the principles of toxic pharmacokinetics (ADME), the TCMSP [8] (https://tcmspw.com/tcmspsearch.php) database was used according to the conditions of oral bioavailability (OB) ≥ 30 and drug-like likeness (DL) ≥ 0.18. The active ingredients of QSDH drug formulary detected by UHPLC-QE-MS were screened. The targets corresponding to the chemical composition of the active ingredients were retrieved through the TCMSP database, and the target names were transformed to gene abbreviations through the UniProt (https://www.uniprot.org/) database [9].

### 2.5. Screening of Targets Related to MG

Using the keyword “myasthenia gravis,” enter the Genecards database (https://www.genecards.org/, updated on June 2, 2021) [10]. DrugBank database (https://www.drugbank.ca/, updated on May 3, 2021) [11]. DisGeNET database (https://www.disgenet.org/, updated on August 16, 2021) [12]. TTD database (http://db.idrblab.net/ttd/updated on June 1, 2021) [13]. PharmGKB database (https://www.pharmgkb.org/, updated on October 4, 2021) [14]. Take the intersection and remove duplicate targets to obtain disease targets, input them into the UniProt database to get the corresponding gene names, and finally get all myasthenia gravis disease-related targets.

### 2.6. Construction Network of the “TCM Active Ingredients - Intersection Target - Disease.”

In order to determine the MG target of the QSDH drug formulary, the intersection target of the MG target and the corresponding target of the QSDH drug formulary active compound were obtained by Perl language analysis, and a Wayne diagram was drawn. The data of the intersection targets of the active compounds and their interactions were imported into Cytoscape3.8.2 software to construct a network of “TCM active components-intersectiontarget-disease,” and topological analysis was conducted to explore the pharmacological mechanism of the active components of QSDH prescription.

### 2.7. QSDH Drug Formulary Predictive Target Protein Interaction (PPI) Network Construction and Analysis

After importing the intersection targets obtained above into the STRING database (https://www.string-db.org) [15], multiple proteins were selected to constrain *Homo sapiens*, hide-free nodes, and retain default values for other parameters to obtain the intersection target interaction network. At the same time, the data were set with medium confidence >0.4 to ensure the reliability of the analysis.

### 2.8. GO and KEGG Enrichment Analysis

The intersection target was imported into the Hiplot database (https://hiplot.com.cn/, updated on May 4, 2021), and the species was *Homo sapiens*. The threshold *P* < 0.01 and the Gene Ontology, GO enrichment analysis, and KEGG (Kyoto Encyclopedia of Genes and Genomes) pathway enrichment analysis were set. R 4.0.3 software was used to draw bubble charts and bar charts from obtaining the biological process (BP), molecular function (MF), cellular components (CC), and main signal pathways involved in the treatment of MG by QSDH drug formulary.

### 2.9. Molecular Docking Verification of Key Active Ingredients and Core Targets

Based on the above research results, the network analyzer plug-in is used to analyze the network topology structure. Active components with the most significant degree value and the largest number of targets are selected as ligands, and core key genes are screened as receptors by the Cytohubba plug-in. The PubChem database (https://pubchem.ncbi.nlm.nih.gov/) downloads the 3D structure of active ingredients in the PDB database (http://www1.rcsb.org/) to obtain the target protein 3D structure, AutoDockTools1.5.6, AutoDock Vina and PyMOL software were used for molecular structure processing and molecular docking.

## 3. Animal Validation

### 3.1. Experimental Animal Selection

Sixty female SPF Lewis rats, weighing 160∼180 g, aged 6∼8 weeks, were provided by Beijing Viton Lever Experimental Animal Technology Co., LTD., certificate No. SCXK (Beijing) 2016–0006. They were fed in the SPF barrier laboratory of the Animal Experiment Center of Changchun University of Chinese Medicine with free drinking water, indoor keep (21 ± 2)°C, relative humidity 50%–60%, and day and night alternating light and dark for 12 h. The experimental procedure was reviewed and approved by the Experimental Animal Ethics Committee of the Changchun University of Chinese Medicine (NO.2021228).

### 3.2. Rat Model Preparation and Grouping

Eight of 60 Lewis female rats were chosen randomly as the adjuvant control group, and the remaining 52 were referred to Baggi et al [16]. The EAMG rat model was established by the active immunization method. Each rat was injected with 200 *μ*L of mixed emulsion, and R*α*97-116 peptide dry powder and *Mycobacterium tuberculosis* H37RA powder were added to PBS. Each 200*μ*L of PBS contained 50 *μ*g of R*α*97-116 dry powder and 1 mg of H37RA dry powder, and the peptide and dry powder were dissolved by ultrasonication on an ice box to make the peptide and dry powder fully fused in PBS until the solution was cloudy. The dissolved liquid is extracted into a 1 mL syringe. Another syringe extracts 200 *μ*L of CFA, and a medical hose connects the two syringes to mix the PBS and CFA of the meaning peptide segment and bacteriophage powder to prevent protein denaturation, the whole process is carried out on the ice, repeatedly pumping two syringes back and forth until milky white liquid appears and the emulsion does not dissolve and does not spread, keeping the spherical shape floating on the water surface for a long time, then the mold-making drug is drug formularyted successfully. The equipped mold-making antigen emulsion was injected subcutaneously at five sites: the tail's base, both hind limbs' foot pads, and both sides of the back. The adjuvant control group was injected with a mixture of CFA and PBS in equal amounts, and the day of the first immunization was recorded as day 0. One booster immunization was given on day 30 and day 45, with the same preparation, injection volume, injection sites, and injection method as the first immunization, except that CFA was replaced with IFA. The adjuvant control group was injected with a mixture of IFA and PBS, with the same injection volume, injection sites, and injection method as the first immunization. The dose, injection site, and injection method were the same as the first immunization. The model was evaluated on day 60, and the change in body mass assessed the success, Lennon's clinical symptom grading method was used [17], and the difference in the level of AChR-Ab in the peripheral serum of the tail vein of rats. Furthermore, Lewis rats meeting the EAMG criteria were divided randomly into eight rats in the model group, the QSDH drug formulary low-dose group (4.8 g/kg/d), the QSDH drug formulary medium-dose group (9.6 g/kg/d), the QSDH drug formulary high-dose group (19.2 g/kg/d), and the prednisone acetate tablet group (5.4 mg/kg/d).

### 3.3. Western Blot

The removed rat spleen tissue was cut and put into RIPA lysis solution, lysed on ice for 30 min at 12000 r/min, centrifuged at 4°C for 10 min, the supernatant was taken, the protein concentration was measured by BCA method, the sample protein concentration was adjusted to the same, the protein samples were separated by 10% sodium dodecyl sulfate-polyacrylamide gel electrophoresis (SDS-PAGE), and then transferred to polyvinylidene fluoride (PVDF) membranes. After the end of the transfer, PI3K, p-PI3K, Akt, p-AKT primary antibodies (1 : 500), and secondary antibodies were incubated with 5% skimmed milk for 1 hour at room temperature. The transferred protein strips were developed by exposure using a chemiluminescence imaging system, and the images were acquired by quantitative optical density analysis.

### 3.4. Statistical Analysis

GraphPad Prism 5.0 software was used for statistical analysis, and measures were expressed as mean plus or minus standard deviation ($\bar{x}$ ± *s*) using one-way ANOVA. *P* < 0.05 indicates that the difference is statistically significant.

## 4. Results

### 4.1. UPLC-QE-MS Detection for QSDH Drug Formulary

A total of 930 compounds in 60 categories, including quinones, terpenoids, flavonoids, and phenols, were detected by UPLC-QE-MS. The corresponding pharmacokinetic parameters (ADME) were searched and downloaded from the TCMSP website. According to OB ≥ 30% and DL ≥ 0.18 as screening conditions, 85 active compounds were obtained, as shown in Table 1.

### 4.2. Screening for Targets Related to MG

**Figure 2:**
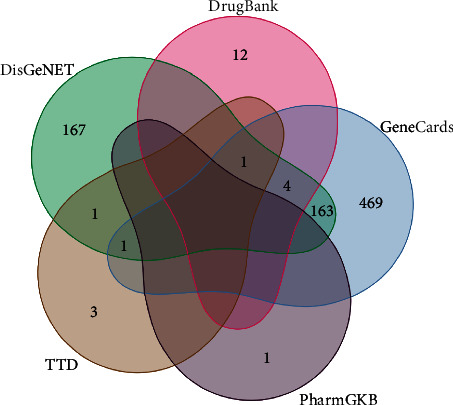
All targets related to MG in various databases.

A total of 822 targets related to myasthenia gravis were obtained from the GeneCards, Drugbank, DisGeNET, TTD, and PharmGKB databases using the keyword “myasthenia gravis,” as shown in Figure 2.

### 4.3. Construction of the “TCM Active Ingredients - Intersection Target - Disease Network”

**Figure 3:**
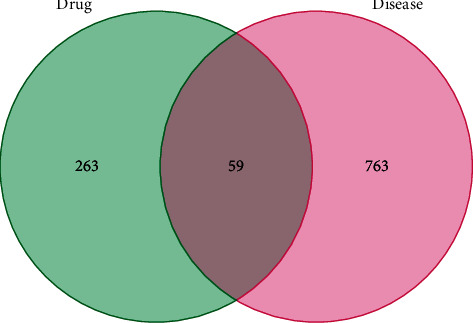
Venn diagram of common targets of TCM active ingredients and diseases.

**Figure 4:**
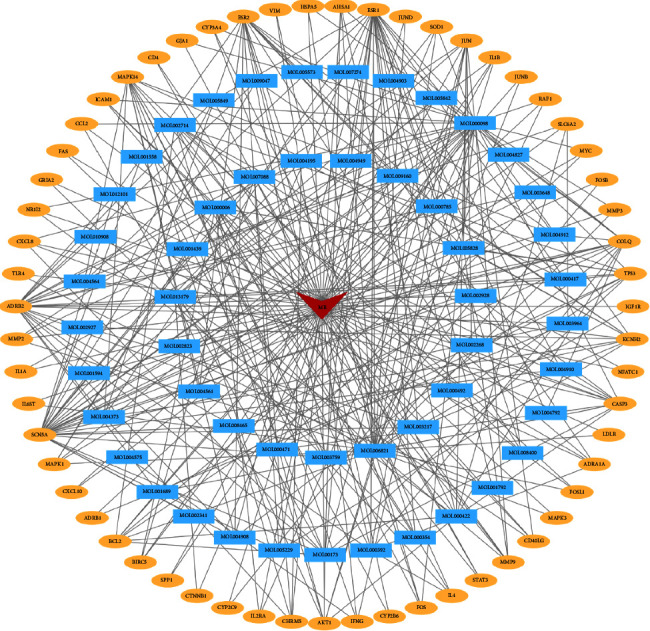
QSDH drug formulary active ingredients-intersectiontarget-disease network. (Red triangles represent diseases, blue rectangles represent active ingredients, and yellow ellipses represent intersection targets).

Ased on Perl language analysis of 85 targets of active components of QSDH prescription and corresponding targets of MG disease, 59 intersection target proteins were obtained, considered as targets of QSDH drug formulary for MG treatment, as shown in Figure 3. Through Cytoscape3.8.2, the “TCM active component-intersectiontarget-disease” network is constructed (Figure 4). The network has 112 nodes and 320 edges. The degree value represents the number of edges connected to this point. The larger the degree value is, the more extensive the effect of this node on other nodes is. The red triangle represents MG disease, the blue rectangle represents active chemical components of the QSDH drug formulary, and the orange ellipse represents the intersection target of active components of herbs and MG. Further topological analysis (based on degree and betweenness centrality of network nodes) shows that the key chemical components in this network are quercetin (MOL000098) and epigallocatechin-3-gallate (MOL006821), luteolin (MOL000006), wogonin (MOL000173), kaempferol (MOL000422), and fisetin (MOL013179). This disease-TCM active ingredient-target network is characterized by complex components, multiple targets, and close interaction between components and targets, forming a complex network for the QSDH drug formulary treatment of MG.

### 4.4. Construction and Analysis of a PPI Network of Predictive Targets for MG Treatment with the QSDH Drug Formulary

**Figure 5:**
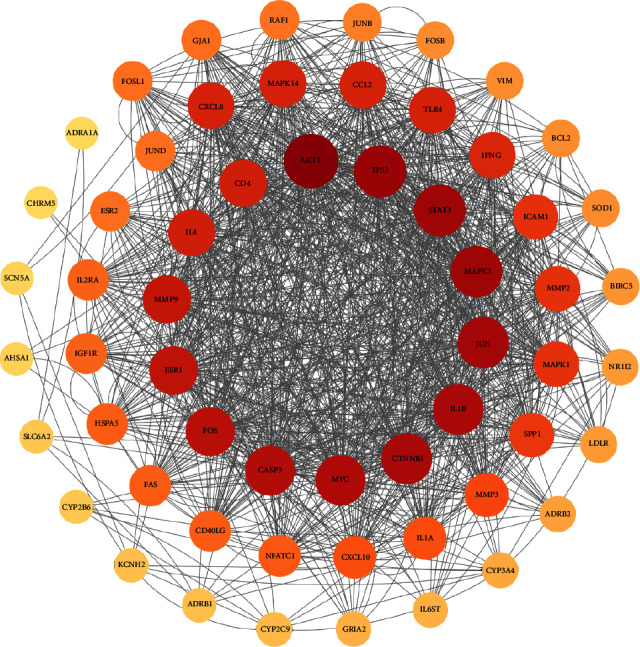
Potential targets of action of QSDH drug formulary for MG treatment PPI network. (Lighter to darker colors and smaller to larger circles represent larger target protein values, representing more important core targets).

**Figure 6:**
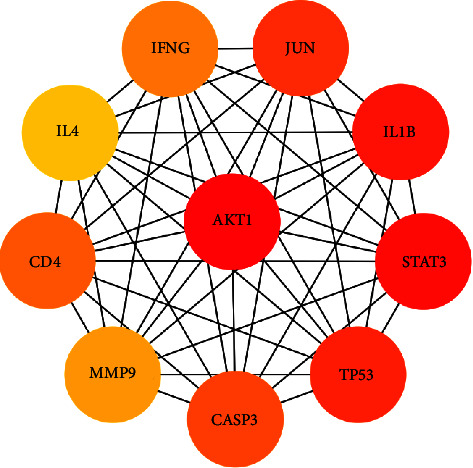
Potential core targets of QSDH drug formulary for MG. (Red represents the most core targets).

To better explain the mechanisms by which the QSDH drug formulary acts concerning MG, we further evaluated its 59 intersecting targets to analyze their relationship. The 59 intersecting targets were imported into the STRING database to obtain protein interaction information, which was visualized using Cytoscape 3.8.2 software (Figure 5). There are 58 nodes and 1172 edges in the intersection target network. The nodes represent the targets of the active ingredient of the QSDH drug formulary for the treatment of MG, and the larger the mutuality value between the targets, the darker the color of the nodes, indicating the more critical the nodes are. The CytoNCA plug-in was used further to perform core screening by degree centrality (DC), closeness centrality (CC), and betweenness centrality (BC) and to construct a protein interaction network by degree value, see Figure 6, to obtain the final core targets: AKT1, STAT3, IL1B, and TP53. They suggested that the QSDH drug formulary may exert the therapeutic effect of MG by regulating these proteins.

### 4.5. GO Enrichment Analysis

**Figure 7:**
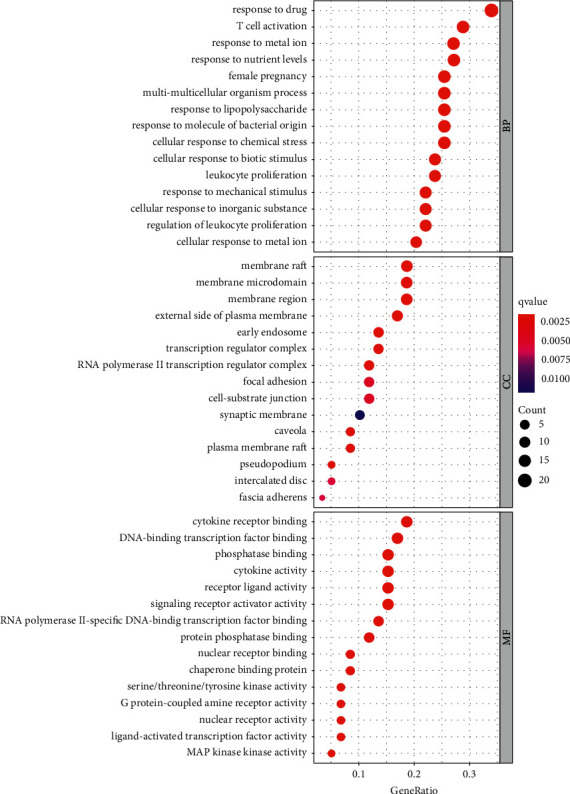
GO enrichment analysis of potential targets of QSDH drug formulary. (The color changes from blue to red, representing smaller P(q.value) values and higher enrichment, and the larger the number of dots, the higher the number of genes, representing higher enrichment).

The 59 intersecting targets were entered into the Hiplot online database, set *p*-value<0.01 and q-value<0.05, The top 10 entries obtained from GO enrichment analysis were performed, and bubble plotted using R4.0.3 software. A total of 1959 items were significantly enriched (*P* < 0.05), of which 1834 were enriched in biological process (BP), 35 in cellular component (CC), and 90 in molecular function (MF), Figure 7. It is mainly related to T cell activation, nutrition level reaction, response to lipopolysaccharide, chemical stress reaction, physical stimulation, oxidative stress, cytokines receptors, cytokine activity, G protein-coupled receptor of neurotransmitter receptor activity, passing the gated ion channel activity involved in the regulation of the postsynaptic membrane potential, and neurotransmitter receptors postsynaptic activity.

### 4.6. KEGG Signaling Pathway Enrichment Analysis

**Figure 8:**
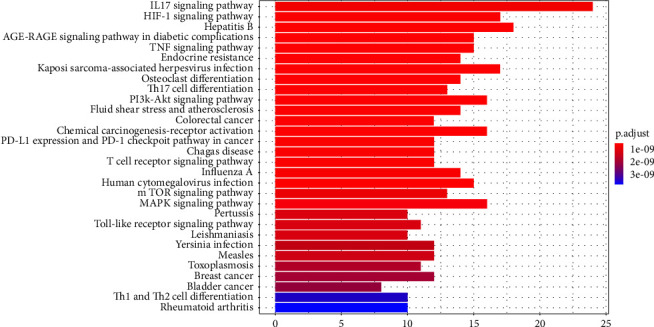
Enrichment analysis of KEGG pathways for potential targets of QSDH drug formulary. (The color changes from blue to red, which means the *P*−value is getting smaller, and the enrichment level is getting higher. The higher the number of genes, the longer the column shape, and the higher the enrichment).

KEGG signaling pathway enrichment analysis of 59 intersecting targets was performed by Hiplot online database, and the results showed that they were mainly enriched in 157 signaling pathways (*P* < 0.05), and the top 30 pathways were bar charted by R 4.0.3 software (Figure 8). The signaling pathways closely related to MG mainly included the IL-17 signaling pathway, the HIF-1 signaling pathway, the PI3K/AKT signaling pathway, and the mTOR signaling pathway. The above results suggest that QSDH can be used to treat myasthenia gravis by regulating multiple biological processes and the coordinated action of multiple signaling pathways.

### 4.7. Molecular Docking Verification

**Figure 9:**
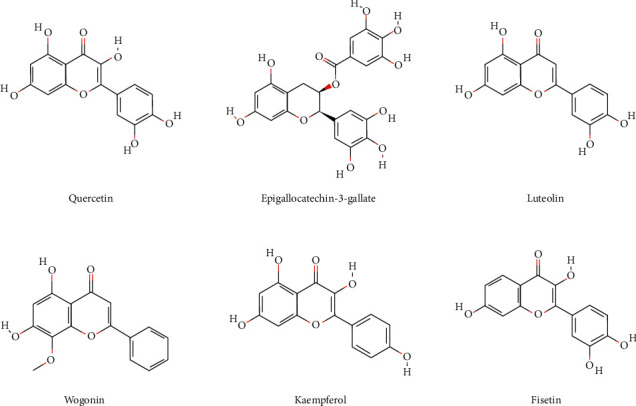
Structural drug formulary of the main active ingredients of QSDH drug formulary.

**Figure 10:**
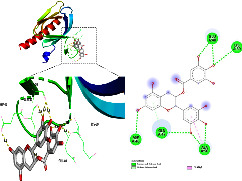
Docking conformation of AKT1 with epigallocatechin-3-gallate.

Six central herbal core ingredients in the QSDH drug formulary were quercetin, epigallocatechin-3-gallate, luteolin, wogonin, kaempferol, fisetin, and epigallocatechin-3-gallate, respectively, according to the “active ingredient-intersectiontarget-disease” network analysis. Their binding ability was predicted with AKT1, the top core target screened by the CytoNCA plug-in. See Figure 9 for the chemical structure of the core active components of herbal medicine. Binding energy < −4.25 kcal/mol indicates some binding activity between the small ligand molecule and the receptor protein, binding energy < −5.0 kcal/mol indicates good binding activity between the two, and binding energy < −7.0 kcal/mol indicates that the ligand has binding activity with the receptor binding solid activity [18]. It was suggested that the more stable the binding conformation of the small molecule receptor to the ligand, the lower its energy and the greater the interaction produced [19]. According to the molecular docking binding energy scores (Table 2), it is clear that the binding energy of quercetin, luteolin, wogonin, kaempferol, fisetin, and the above five compounds with the critical core action target AKT1 are all less than −5.0 kcal/mol, indicating an excellent bonding activity between them. The binding energy of AKT1 to epigallocatechin-3-gallate was less than /−7.0 kcal/mol, indicating a solid binding activity between the two. Molecular docking visualization was performed separately using Pymol 2.4.2 software (Figure 10). Epigallocatechin-3-gallate formed hydrogen bonds with five amino acids, ASP-46, GLU-40, LYS-39, ALA-50, and GLN-47, near the active site to bind to AKT1.

### 4.8. Evaluation of EAMG Rat Models

#### 4.8.1. Lennon Clinical Score

**Figure 11:**
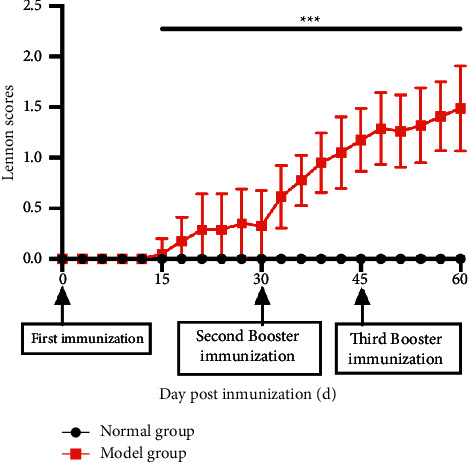
Lennon's clinical score changes in the EAMG model and adjuvant control group (^*∗∗∗*^*P* < 0.001).

As depicted in Figure 11, during the first 15d after the first immunization, there was still no significant change in Lennon's clinical symptom score. However, the EAMG-modeled rats began to show a gradual decrease in grip strength and activity. From the 15th day after the first immunization, the rats in the model group began to show significantly weaker bilateral forelimb grasping power, reduced antagonism, less flexible body than the adjuvant control group, and preferred to curl up in the corner of the cage. After the second booster immunization (the 30th day after the first immunization), the EAMG symptoms gradually increased (*P* < 0.001).

#### 4.8.2. Changes in Serum AChR-Ab of EAMG Model Rats

**Figure 12:**
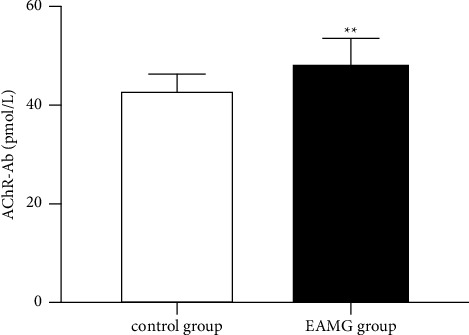
Comparison of serum AChR-Ab levels in EAMG-modeled rats and adjuvant control rats (^*∗∗*^*P* < 0.01).

On day 60 after the first immunization, the serum AChR-Ab levels of rats were analyzed by ELISA. As shown in Figure 12, the serum AChR-Ab levels of rats in the EAMG modeling group were significantly higher than those in the adjuvant control group (*P* < 0.01).

### 4.9. Effect of QSDH Drug Formulary on EAMG Rats

#### 4.9.1. Effect of QSDH Drug Formulary on Lennon's Clinical Symptom Score in EAMG Rats

**Figure 13:**
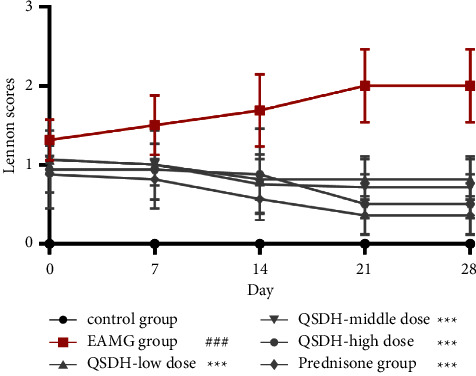
Effect of QSDH drug formulary on Lennon's clinical score in EAMG rats. (Compared with the adjuvant control group ^####^*P* < 0.001; compared with the EAMG model group ^*∗∗∗*^*P* < 0.001).

The EAMG model was successfully established and then randomized to group administration of the intervention, with Lennon clinical score tests performed every seven days during treatment. During the administration treatment, the clinical symptoms of rats in the EAMG model group did not improve as the experiment progressed. Even individual rats showed signs of paralysis, convulsions, loose skin, jaws clinging to the bottom of the cage, and inability to eat, and the Lennon clinical score was significantly different from that of the adjuvant control rats (*P* < 0.001), as shown in Figure 13. Indicates that the EAMG model was established stably and did not heal during the administration period. As the experiment continued, the clinical symptoms of the rats in the high, medium, and low doses of QSDH drug formulary and prednisone acetate administration groups were gradually relieved, and the Lennon clinical score tended to decrease and was significantly different from that of the EAMG model group (*P* < 0.001).

#### 4.9.2. Effect of QSDH Drug Formulary on Serum AChR-Ab Levels in EAMG Rats

**Figure 14:**
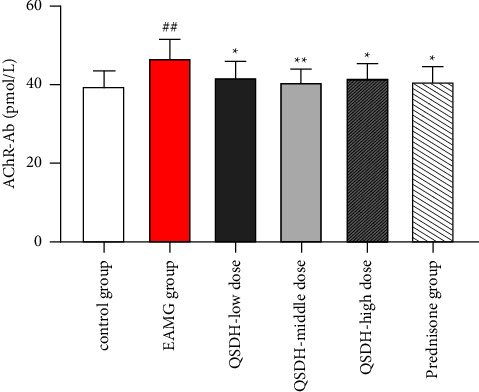
Effect of QSDH drug formulary on serum AChR-Ab levels in EAMG rats. (Comparison with the adjuvant control group: ^##^*P* < 0.01; comparison with the EAMG model group: ^*∗*^*P* < 0.05, ^*∗∗*^*P* < 0.01).

At the end of the dosing cycle, the rats were anesthetized with gas anesthesia apparatus, taken through the abdominal aorta, and the serum was separated by centrifugation. The ELIAS method detected the peripheral serum AChR-Ab content of the rats in each experiment group. The serum AChR-Ab levels of rats in the EAMG model group were significantly higher than those in the adjuvant control group (*P* < 0.01). After the intervention of each dosing group, the serum AChR-Ab levels of rats in the QSDH drug formulary low, medium, and high-dose groups and the prednisone acetate tablet group were significantly reduced compared with those in the EAMG model group (*P* < 0.05, *P* < 0.01, Figure 14).

#### 4.9.3. QSDH can Inhibit the PI3K/AKT Signaling Pathway

**Figure 15:**
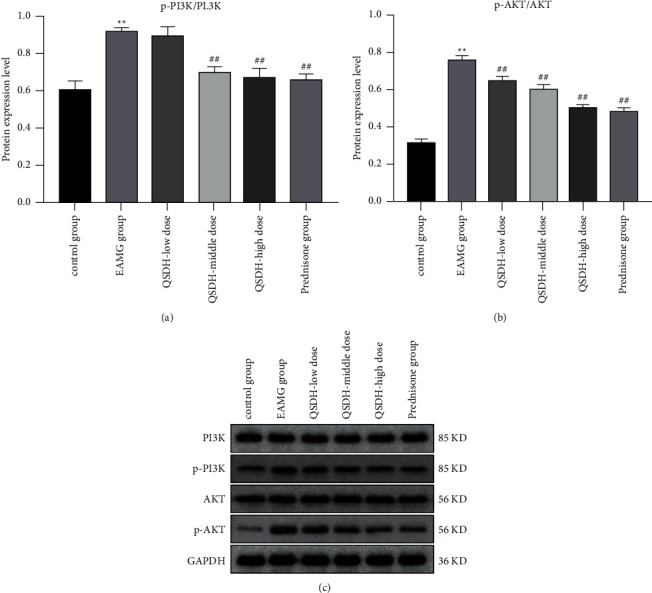
Effect of QSDH drug formulary on the expression level of the PI3K/AKT signaling pathway-related proteins in EAMG rats. (Figure a shows the significant analysis of PI3K and p-PI3K protein expression in the spleen tissues of rats in each experimental group, Figure b shows the significant analysis of AK and p-AKT protein expression in the spleen tissues of rats in each experimental group, and Figure c shows the expression of related proteins in the spleen tissues of rats in each experimental group by Western blot. Comparison of the EAMG model group with adjuvant control group ^*∗∗*^*P* < 0.01. QSDH drug formulary each dose group compared with the EAMG model group #*P* < 0.05, ^##^*P* < 0.01).

Based on the core targets screened by network pharmacology and the results of the KEGG enrichment analysis, we further verified whether the QSDH drug formulary for MG treatment interferes with the PI3K/AKT signaling pathway. Western blot was used to detect the expression of the PI3K/AKT pathway-related proteins in the spleen tissues of each group of rats. Detection of protein expression of PI3K, phosphorylated PI3K (p-PI3K), AKT, and phosphorylated AKT (p-AKT). As Figures 15(a)–15(b) show, the expression of pathway-related proteins p-PI3K and p-AKT in the spleen tissue of rats in the EAMG model group was significantly higher (*P* < 0.01) than in the adjuvant control group. The pathway-related proteins p-PI3K and p-AKT decreased to different degrees after each dose group of QSDH drug formulary low, medium, and high and positive drug treatment (*P* < 0.05, *P* < 0.01).

## 5. Discussion

Network pharmacology is based on systems biology theory, integrates pharmacology, high-throughput sequencing, genomics, and other technologies, emphasizing the multichannel regulation of signaling pathways [20]. Our study explores the mechanism of action and potential therapeutic targets of the QSDH drug formulary for MG from the perspective of network pharmacology. Using ultra-performance liquid chromatography Q Exactive-mass spectrometry, network pharmacology, and molecular docking technology, 930 chemical components were analyzed from the QSDH drug formulary. The “active component-intersectiontarget-disease” network of QSDH drug formulary treatment for MG was constructed. Eighty-five active components and 322 potential targets of quercetin, luteolin, wogonin, kaempferol, fisetin, and epigallocatechin-3-gallate were screened. AKT1, STAT3, IL1B, and TP53 were selected as core targets through the PPI network, and AKT1 was the first and most core target. GO, and KEGG enrichment analysis further explored the biological pathways involved in the expression of potential target proteins, which were enriched in the IL-17 signaling pathway, the HIF-1 signaling pathway, the PI3K-Akt signaling pathway, and the mTOR signaling pathway. PI3K-AKT was the most abundant protein enrichment signaling pathway. The binding activity of the core active ingredient and core target protein in the QSDH drug formulary were verified. Finally, the rat experiment verified that AKT1 might be the main target of QSDH prescription in the treatment of MG, and QSDH prescription can play a role in the treatment of MG by inhibiting the PI3K/AKT signaling pathway.

The active ingredient-intersection target network showed that quercetin, epigallocatechin-3-gallate, luteolin, wogonin, kaempferol, and fisetin matched more targets, which may be the core compounds and essential material basis for the QSDH drug formulary to exert its therapeutic effect on MG. Studies have shown that epigallocatechin-3-gallate, quercetin, luteolin, wogonin, kaempferol, and fisetin have anti-inflammatory, antioxidant, immunomodulatory, and neuroprotective effects [21–26]. Epigallocatechin-3-gallate promotes dendritic growth and synaptogenesis through selective inhibition of the AKT/mTOR signaling pathway, inhibits acetylcholinesterase activity, and effectively ameliorates neurological damage in neurodegenerative diseases. In addition, it can regulate immune and inflammatory responses by influencing various aspects of immune cell function in innate and adaptive immunity. It can affect the differentiation of initial CD4+T cells to different effector subpopulations and thus treats autoimmune inflammatory diseases [27]. Quercetin has a wide range of pharmacological effects, including anti-inflammatory, antiviral, antibacterial, and immunomodulatory effects, and can significantly upregulate IFN-*γ* in Th1 and downregulate IL-4 expression in Th2, thereby regulating immune function [28]. Luteolin has antitumor, anti-inflammatory, and cerebrovascular protective effects, and its related mechanism involves the regulation of PI3K/AKT, MAPK, and other signaling pathways as well as the expression of related cytokines and kinases [29]. An experimental study found that kaempferol acts the same way as the immunosuppressant cyclosporine A, mediating calcium-regulated neural phosphatase activity from inhibiting immune cell differentiation [30]. Fisetin reduces IL-17 production by CD4+T lymphocytes through inhibition of the PI3K/AKT/mTOR pathway and decreases Th1/Th17 pro-inflammatory cytokine secretion in human peripheral blood mononuclear cells [31]. The above can indicate that the active ingredients in the QSDH drug formulary can regulate the subpopulation differentiation of CD4+T cells, alter the secretion of related cytokines between Th1/Th2/Th17, and exert anti-inflammatory, immunomodulatory, and neurological function protection effects.

The core targets obtained from screening intersecting targets in the PPI network by Cytohubba and CytoNCA plug-ins, of which the top three occupied by AKT1, STAT3, and TP53, were predicted to be the possible main targets of action of the QSDH drug formulary for the treatment of MG. Studies have reported that AKT1 enhances the differentiation of CD8+T cells, improves the proliferation of CD8+T cells, and inhibits the over-activation of DCs, thus regulating the immune response [32]. The study showed increased expression of Th1 and Th17 cells and their associated cytokines IL-1, IL-6, IL-17, IFN-*γ*, and TNF-*α* in the peripheral serum of MG patients [33]. The expression of IL-6 can be regulated by inhibiting the AKT/mTOR signaling pathway to improve the symptoms of muscle fatigue weakness in MG patients [34]. STAT3, which has immunomodulatory effects, modulates the production of Th1 cell-specific cytokines by altering the balance between Th1 and Th2 cells, thereby altering immune function and inflammatory responses [35]. TP53 is currently the most widely studied oncogene. TP53 can be both an activator and an inhibitor of autophagy. Under stress conditions such as nutrient depletion or hypoxia, TP53 promotes autophagy activation by inhibiting the mTOR signaling pathway, thereby suppressing the inflammatory response. Inhibition of TP53 increases IL-6 gene expression while promoting cell proliferation and differentiation [36].

Based on the KEGG pathway enrichment analysis results, we can see that the QSDH drug formulary exerts its effect on the treatment of MG mainly through signaling pathways such as the IL-17 signaling pathway, the HIF-1 signaling pathway, the PI3K-AKT signaling pathway, and the mTOR signaling pathway. The PI3K-AKT signaling pathway regulates cell proliferation, differentiation, apoptosis, and glucose transport and can regulate T cells' development, stability, and function [37]. Studies suggest that PI3K-AKT-specific inhibitors significantly upregulate Th1 and Th17 cells in PBMCs of MG patients, and mTOR/HIF-1*α* activates the expression of glycolytic genes to provide energy for rapid activation of immune cells, suggesting that the PI3K/AKT signaling pathway regulates immune function in MG [38]. Our in vivo experimental study in rats confirmed that the QSDH drug formulary could alleviate the symptoms of EAMG and reduce the level of acetylcholine receptor antibodies in the peripheral blood of EAMG rats by inhibiting the PI3K/AKT signaling pathway, thus demonstrating that the QSDH drug formulary for MG can work by inhibiting the PI3K/AKT signaling pathway.

We selected the core active ingredients quercetin, epigallocatechin-3-gallate, luteolin, wogonin, kaempferol, and fisetin as ligands and the first core target AKT1 as the receptor for molecular docking. Furthermore, we verified the pharmacodynamic substance basis of the QSDH drug formulary for MG treatment. The results showed that the main active compounds could stably bind to the core target AKT1 through hydrogen bonding. Among them, epigallocatechin-3-gallate formed the strongest hydrogen bond to AKT1 with asp-46, Glu-40, LYS-39, AlA-50, and GLN-47 amino acids near the active site. It further indicates that the QSDH drug formulary can treat myasthenia gravis by acting on the AKT1 target protein.

## 6. Conclusions

In conclusion, 85 active pharmaceutical ingredients of the QSDH drug formulary were identified by UHPLC-QE-MS combined with the network pharmacology method. Preliminary prediction QSDH drug formulary can regulate the PI3K/AKT signaling pathway through quercetin, epigallocatechin-3-gallate, luteolin, wogonin, kaempferol, fisetin, and other active ingredients, acting on AKT1 target protein to inhibit inflammation, immune function, and cell metabolism, to achieve the purpose of treating MG. Provides new insights into the QSDH drug formulary's mechanism to alleviate MG disease's progression.

## Figures and Tables

**Table 1:** Active ingredients of QSDH drug formulary.

Mol ID	Molecule Name	OB (%)	DL
MOL000471	Aloeemodin	83.38	0.24
MOL011578	Bilobalide	84.42	0.36
MOL000354	Isorhamnetin	49.6	0.31
MOL000417	Calycosin	47.75	0.24
MOL000471	Emodin	83.38	0.24
MOL002928	Oroxylin a	41.37	0.23
MOL005573	Genkwanin	37.13	0.24
MOL002714	Baicalein	33.52	0.21
MOL001689	acacetin	34.97	0.24
MOL000422	Kaempferol	41.88	0.24
MOL005842	Pectolinarigenin	41.17	0.3
MOL001942	Isoimperatorin	45.46	0.23
MOL003626	Ostruthin	30.65	0.23
MOL000006	Luteolin	36.16	0.25
MOL002341	Hesperetin	70.31	0.27
MOL000392	Formononetine	69.67	0.21
MOL003648	Inermin	65.83	0.54
MOL001297	Eicosenoic acid	30.7	0.2
MOL002881	Flavone base + 3O, 1MeO	31.14	0.27
MOL000098	Quercetin	46.43	0.28
MOL004800	Pelargonidin-3-O-glucoside	38.72	0.71
MOL004903	Liquiritin	65.69	0.74
MOL007274	Cirsimaritin	30.35	0.3
MOL009047	Eudesmin	33.29	0.62
MOL012101	5-hydroxy-6,7-dimethoxyflavone	34.04	0.26
MOL004373	Icaritin	45.41	0.44
MOL005320	Arachidonic acid	45.57	0.2
MOL005190	Eriodictyol	71.79	0.24
MOL008845	Deoxycholic acid	40.72	0.68
MOL004792	Nodakenin	57.12	0.69
MOL004425	Icariin	41.58	0.61
MOL003217	Isoxanthohumol	56.81	0.39
MOL002268	Rhein	47.07	0.28
MOL002776	Baicalin	40.12	0.75
MOL009160	Loureirin A	40.43	0.19
MOL005849	Didymin	38.55	0.24
MOL003378	Demethylwedelolactone	33.94	0.43
MOL004564	Kaempferid	73.41	0.27
MOL002823	Herbacetin	36.07	0.27
MOL004575	Astilbin	36.46	0.74
MOL003908	Strophanthidin	99.94	0.78
MOL004908	Glabridin	53.25	0.47
MOL003759	Iristectorigenin B	63.36	0.34
MOL000492	Cianidanol	54.83	0.24
MOL008698	Dihydrocapsaicin	47.07	0.19
MOL000492	Deoxynivalenol	54.83	0.24
MOL005828	Nobiletin	61.67	0.52
MOL001558	Sesamin	56.55	0.83
MOL002927	Skullcapflavone II	69.51	0.44
MOL004561	Sudan III	84.07	0.59
MOL000173	Wogonin	30.68	0.23
MOL000392	Formononetin	69.67	0.21
MOL010485	Eicosapentaenoic acid	45.66	0.21
MOL001714	5,9-dimethyltetracyclo-dicarboxylic acid	59.94	0.86
MOL000785	Palmatine	64.6	0.65
MOL003950	1-methyl-2-[(6Z)-6-undecen-1-yl]-4(1H)-quinolinone	48.48	0.27
MOL001439	Arachidonic acid (not validated)	45.57	0.2
MOL005658	Periplogenin	36.61	0.74
MOL004759	Napelline	34.48	0.72
MOL002397	Karakoline	51.73	0.73
MOL010908	Lindenenol	52.05	0.18
MOL001594	Pisatin	88.05	0.64
MOL007179	Linolenic acid ethyl ester	46.1	0.2
MOL010861	Vitamin D3	45.66	0.48
MOL010828	Cynaropicrin	67.5	0.38
MOL007077	Sclareol	43.67	0.21
MOL008465	Hirsutine	32.75	0.64
MOL001792	Liquiritigenin	32.76	0.18
MOL006821	Epigallocatechin-3-gallate	55.09	0.77
MOL004058	Khellin	33.19	0.19
MOL008400	Glycitein	50.48	0.24
MOL003964	1-methyl-2-undecylquinolin-4-one	47.59	0.27
MOL004827	Semilicoisoflavone B	48.78	0.55
MOL004195	Corydaline	65.84	0.68
MOL013179	Fisetin	52.6	0.24
MOL013276	Poncirin	36.55	0.74
MOL005229	Artemetin	49.55	0.48
MOL005922	Acanthoside B	43.35	0.77
MOL011616	Fortunellin	35.65	0.74
MOL000546	Spirost-5-en-3-ol, (3beta, 25R)-	80.88	0.81
MOL004910	Glabranin	52.9	0.31
MOL004912	Glabrone	52.51	0.5
MOL004949	Isolicoflavonol	45.17	0.42
MOL000830	Alisol B	34.47	0.82
MOL007088	Cryptotanshinone	52.34	0.4

**Table 2:** Molecular docking score.

Core Targets	PDB ID	Compound Name	Binding energy
AKT1	P31749	Quercetin	−6
		Luteolin	−6.3
		Wogonin	−6
		Kaempferol	−5.9
		Fisetin	−6
		Epigallocatechin-3-gallate	−7.6

## Data Availability

The datasets used and/or analyzed during the current study are available from the corresponding author on reasonable request.
